# Advancements in macrophage research for cardiovascular disease

**DOI:** 10.3389/fphys.2025.1647865

**Published:** 2025-08-14

**Authors:** Meiling Cao, Yu Sun, Xinyue Zhang, Jiahui Zou, Mingyue Shi, Lei Li, Hongkun Jiang

**Affiliations:** ^1^ Department of Neonatology, The First Hospital of China Medical University, Shenyang, China; ^2^ Department of Pediatrics, The First Hospital of China Medical University, Shenyang, China; ^3^ Department of Pediatrics, Liaoning Provincial People’s Hospital, Shenyang, Liaoning, China; ^4^ Department of Orthopaedic Surgery, Shengjing Hospital of China Medical University, Shenyang, Liaoning, China

**Keywords:** atherosclerosis, cardiovascular disease, cell metabolism, hypertension, macrophage, mechanism of action, myocardial infarction

## Abstract

Cardiovascular disease is a major cause of human morbidity and mortality. With the rising prevalence of cardiovascular diseases and their increasing economic impact on individuals and society, a deeper understanding of their pathogenesis is essential. Macrophages play the crucial role in this regulatory network as key cell types in the innate immune system. This review provides an overview of the subpopulations, heterogeneity, and ontogenetic diversity of macrophages. It emphasizes how various cellular metabolic processes influence the biological functions of macrophages. Additionally, it explores the mechanisms through which macrophages function in different cardiovascular diseases (e.g., atherosclerosis, hypertension, or myocardial infarction) and examines their potential therapeutic applications. The review also addresses the current limitations and future directions for research and therapeutic strategies involving cardiac macrophages in cardiovascular diseases.

## 1 Introduction

Cardiovascular diseases, encompassing conditions such as atherosclerosis, and heart failure, significantly contribute to human morbidity and mortality due to a combination of environmental and socio-economic pressures (Osborne et al., 2020; Li et al., 2024; Christ et al., 2024; Khan et al., 2022). Globally, around 17.9 million people die from cardiovascular diseases annually (Meier et al., 2019). Macrophages are renowned for their heterogeneity and diversity,and they play a crucial role in both physiological and pathological processes of the cardiovascular system.

As the important part of the regulation process, macrophages can timely monitor and respond to various pathogens and environmental stimuli, maintain the homeostasis of tissues and organs, regulate the induction and immune response of pathogen infection, and promote tissue repair and remodeling of tissue damage during tissue development (Epelman et al., 2014a).

In the late 19th century, Ilya Metchnikoff first discovered macrophages, conserved phagocytes that have evolved over 500 million years (Tauber, 2003; Cooper and Alder, 2006). Later, for more than 40 years, Van Furth and Cohn proposed that all tissue macrophages originated from monocytes in circulating blood and were considered to be an important part of mononuclear phagocytes (van Furth and Cohn, 1968). However, with the development of scientific research, people have a more accurate understanding of the origin of macrophages. It has been found that most adult tissue macrophages do not originate from blood-monocytes, but from the early embryonic development process, and embryonic hematopoiesis can differentiate macrophage subsets in tissues (Epelman et al., 2014b). There is evidence suggesting that most adult tissue-resident macrophages originate from the yolk sac during embryonic development and are monocytes with the ability of self-renewal and the capacity to independently maintain themselves (Epelman et al., 2014b; Davies et al., 2013; Hashimoto et al., 2013). Moreover, the macrophages in each organ are derived from either the embryo or the adult and have their own unique combination patterns. This indicates that some tissue-resident macrophages in patients are largely unaffected, and having a monocytopenia is further evidence supporting this assumption (Hashimoto et al., 2013). Monocytes, as a highly plastic and dynamic cell system, can complement the classic tissue-resident mononuclear phagocytes. These cells derived from monocytes serve as short-lived effector cells and play various roles within the tissues. Embryonic macrophages participate in tissue remodeling, while adult-derived macrophages mainly assist the host in defense. In addition to these differences, it has also been observed that embryonic and adult-derived macrophages coexist in many different organs (Epelman et al., 2014b; Davies et al., 2013; Hashimoto et al., 2013; Galli et al., 2011; Varol et al., 2015).

The efficient phagocytosis of macrophages on apoptotic cells is called efferocytosis (Zuttion et al., 2024).In the classic subgroup classification of Macrophages 1 (M1)/2 (M2), M1 represents “pro-inflammatory” Macrophages and M2 represents “anti-inflammatory” macrophages (Wang et al., 2019).

Macrophages play the pivotal role in both the development and treatment of cardiovascular diseases. For instance, the lipopolysaccharide-induced polarization of macrophages toward the M1 phenotype contributes to the reduction of systemic inflammation (De Bartolo et al., 2024). Quercitrin (Que) is a common flavonoid in fruits and vegetables that has antioxidant, anti-inflammatory, antitumor and other effects (Varol et al., 2015).Quercetin enhances cardiac remodeling after myocardial infarction by decreasing glycolysis, increasing organophosphorylation in tissues, and altering arginine metabolic pathways, which shifts macrophages from the M1 to the M2 phenotype (Liu et al., 2024).

Various cutting-edge therapies, such as exploring pro-inflammatory mechanisms *in vitro* and creating new immunizations, have been utilized in the manage cardiovascular and metabolism-related diseases (Tylek et al., 2024; Fernandez et al., 2019). Nevertheless, the utilization of macrophages in cardiovascular disease treatment faces constraints. These limitations stem from an inadequate comprehension of the metabolic pathways specific to macrophages and concerns related to the safety and invasiveness associated with these therapeutic approaches (Liu et al., 2022).

Therefore, this review provides a comprehensive overview of macrophages, discussing their subpopulations, heterogeneity, and ontogenetic diversity. We highlight the essential roles of macrophages in preventing tissue inflammation and necrosis, and in maintaining tissue homeostasis through efferocytosis, immunity, secretion, and metabolism. Additionally, we explore how macrophages are utilized in treating cardiovascular diseases, focusing on their therapeutic effects involving cytokines, signal transduction, gene expression regulation, and interactions with other cells. We also address the current limitations and future prospects of macrophages in the research and application of therapeutic strategies for cardiovascular diseases.

## 2 Macrophages in cardiovascular system

**FIGURE 1:**
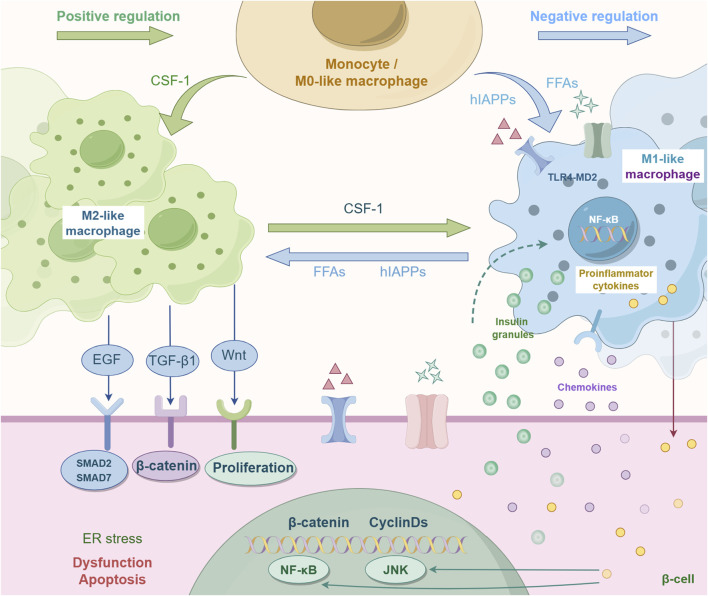
Mutual polarization of macrophage M1-type and M2-type. Traditionally, macrophages are classified into two subtypes: M1 and M2. These cells can dynamically switch between these states in response to specific conditions (such as microbial stimulation), and this process is known as macrophage polarization. This process is regulated by multiple factors. For instance, CSF-1 is a key factor promoting M2 polarization. CSF-1 can reduce the expression of M1-related markers by inhibiting the NF-κB pathway (the core pathway of pro-inflammatory signals). FFAS and hlAPPs can inhibit M1 polarization. EGF, TGF-β1, and Wnt can regulate the function of macrophages and promote M2 polarization, etc.

Macrophages are highly adaptable. Traditionally categorized into M1 and M2 subtypes, these cells exhibit dynamic transitions between these states in response to specific conditions, such as microbial stimulation (Figure 1) (Wang et al., 2019).

Macrophage polarization is not fixed, but can be accomplished by receiving and integrating various signals, such as microorganisms, tissue cell damage, death, and normal tissue microenvironment (Yunna et al., 2020). There are three different ways to control polarization: 1) epigenetic effects on macrophage activity (Chawla, 2010); 2) external inflammatory products and cytokines; 3) The internal organization of the normal microenvironment (Luo et al., 2024). M1 is mostly polarized by lipopolysaccharide (LPS) and Th1 cytokines (IFN-γ) (Lee et al., 2023),while M2 is mostly polarized by Th2 cytokines (such as IL-4 and IL-3) to produce anti-inflammatory factors, which are closely related to immune regulation, tissue repair and healing (Duluc et al., 2009).

However, recent findings indicate that this traditional M1/M2 dichotomy is limited, revealing greater heterogeneity and more complex phenotyping of macrophages than has been previously understood (Zhang X. et al., 2024).

Within the human heart, diverse macrophage populations exist, each with distinct origins and functional roles. Hulsmans’ team used single-cell sequencing, and identified that the overall mouse cardiac macrophage population encompasses CCR2+MHCII^high^, CCR2-MHCII^high^, and CCR2-MHCII^low^ subpopulations (Hulsmans et al., 2017). Bajpai et al. discovered that the human myocardium includes distinct subpopulations of CCR2-macrophages, CCR2+ macrophages, and CCR2+ monocytes (Bajpai et al., 2018). In 2020, Nathan R. Tucker et al. employed single-cell sequencing and other methodologies to discern cardiac-resident macrophages. These cells exhibit characteristics, including scavenger receptors such as CD163, the E3 ubiquitin ligase March1, COLEC12, the mannose receptor MRC1, and natural resistance-associated macrophage protein 1 (SLC11A1 or NRAMP1) (Tucker et al., 2020).

In addition, other studies have shown that macrophages can be divided into three LYVE1 macrophage groups, namely, LYVE1 MP1, LYVE1 MP2 and LYVE1 MP3 (Litviňuková et al., 2020). LYVE1 MP1-2 is rich in clathrin and cathepsin group, while LYVE1 MP3 is rich in HLA-DOA, HLA-DQA1/2 and HLA-DQB1 (Litviňuková et al., 2020). In 2018, Lim HY et al. found that LYVE1 macrophages can maintain vascular homeostasis by regulating the collagen of smooth muscle cells in tissues, and also play a role in white blood cells (Lim et al., 2018), which is closely related to cardiovascular remodeling. Antigen-presenting macrophages include FOLR2−, LYVE, and MERTK, which are enriched in HLA-DMA, HLA-DMB, HLA-DRA, HLA-DPA1, and Trem2. LYVE1 and FOLR2 are expressed by monocyte derived macrophages, and their markers are CEBPB, S100A8, CCL13 and CCL1811. In a recent 2023 study, Julius L Decano et al. analyzed IFN-γ (M (IFN-γ) -activated primary human macrophages using immunoassays and functional tests, single-cell RNA sequencing, time-history cluster proteomics, and metabolite consumption. At least two major macrophage clusters have been found in M (IFNγ) (Decano et al., 2023). Macrophages are important cells involved in and regulating all inflammatory diseases and are very important for their regression.

Cardiac macrophages are immune cells that reside in the heart tissue and belong to the mononuclear-macrophage system. They have unique developmental origins, phenotypic characteristics and functions, and play a crucial role in maintaining cardiac homeostasis, repairing damage and progressing diseases. Efferocytosis resident macrophages play a variety of functions and roles in regulating the physiology and pathophysiology of the cardiovascular system through efferocytosis, immune regulation, autocrine and paracrine, and cell metabolism. For example, antibody blocking of the “Don’t eat me” signal CD47 can enhance the efferocytosis effect, reducing the lesion size and necrotic core, and the cytosomatogenic receptor LRP1 in macrophages is necessary for anti-CD47 blocking (Mueller et al., 2022). Therefore, more understanding of the classification, subpopulation, polarization, phenotype and mechanism of action of macrophages will be conducive to the treatment of various cardiovascular and metabolic related diseases by interfering with macrophages (such as exploring the proinflammatory mechanism *in vitro* and developing novel immune therapies) (Tylek et al., 2024; Fernandez et al., 2019).

## 3 Macrophages in cardiovascular diseases

**FIGURE 2:**
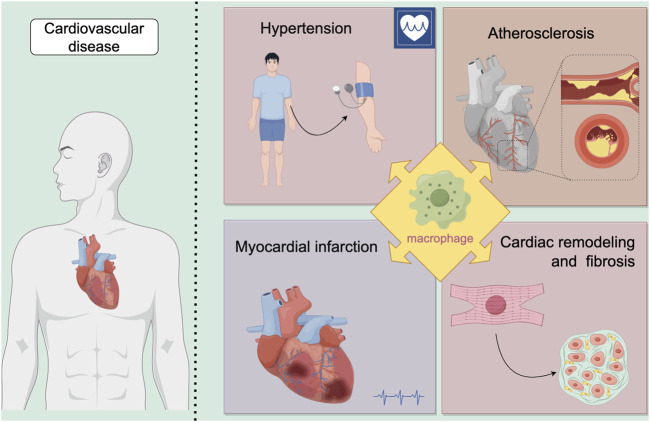
Macrophages play a significant role in various diseases. Our review focuses on their involvement in cardiovascular diseases, specifically examining four key areas: hypertension, atherosclerosis, myocardial infarction, and cardiac remodeling and fibrosis.

Macrophages, a pivotal immune cell type, play essential roles in maintaining tissue homeostasis, regulating inflammation, and promoting wound repair during disease pathogenesis. Within the cardiovascular system specifically, macrophages critically influence disease progression through diverse mechanisms, including immune modulation, mediation of inflammatory processes, participation in tissue repair, and regulation of cell death pathways. This section will focus on elucidating the specific mechanisms by which macrophages contribute to hypertension, atherosclerosis, myocardial infarction, cardiac remodeling and fibrosis.(Figure 2; Table1). This exploration seeks to provide a foundation for understanding their complex regulatory functions in these diseases.

**TABLE 1:** Macrophage subtypes and main function in major cardiovascular diseases.

Disease	Macrophage Subtype	Main Function	Reference
Hypertension	M1 Macrophages	Amplify inflammatory response; Promote vascular dysfunction; Contribute to blood pressure elevation	Fehrenbach and Mattson (2020)
	Infiltrated Macrophages (Predisposed to M1 macrophages)	Dominant population in PVAT microenvironment; Actively promote perivascular adipose tissue (PVAT) remodeling in hypertension	Ye et al. (2019)
	M1 Macrophages	Secrete pro-inflammatory cytokines、Promote fibrosis and catecholamine release	Wei et al. (2021)
	IFNα-dysregulated macrophages/microglia	Mediate cerebrovascular regression via endothelial contact and junctional retraction; Exhibit suppressed innate immunity and vasculo/neuroprotective gene expression	Ying et al. (2021)
	Cardiac Resident Macrophages (RMs)	Maintain reparative gene programs (e.g., Igf1 expression) in homeostasis	Apaydin et al. (2023)
	Hypertension-Activated Cardiac RMs	Drive adaptive cardiomyocyte growth and increased cardiac mass	Apaydin et al. (2023)
	M2 Macrophages	Exert cardioprotective effects by attenuating LVH and fibrosis	Zaman et al. (2021)
Atherosclerosis	Control Macrophages (NCOR1-competent)	Suppress atherogenic processes by restraining PPARγ target genes	Bäck et al. (2019)
	NCOR1-Deficient Macrophages	Drive atherosclerosis progression via enhanced lipid uptake and inflammation	Bäck et al. (2019)
	Control Macrophages	Mediate lipid uptake via LRP5/PCSK9-dependent LDL internalization	Oppi et al., 2020
	PSRC1-Expressing Macrophages	Suppress inflammation and delay AS by sequestering ANXA2 and inhibiting STAT3 activation	Badimon et al. (2021)
	SPP1+ Macrophages	Promote migration and proliferation of fibroadipogenic progenitor cells (FAPCs) via OPN-CD44/integrin interaction, leading to increased fibrosis in coronary PVAT.	Pan et al. (2023)
	Resident Macrophages	Uptake of desialylated LDL leading to fatty streak formation; Initiation of atheromatous plaque development Conversion to lipid-laden cells in aortic root lesions	Linton et al. (2023)
	VSMC-Derived Macrophage-Like Cells	Transform into foam cells via mechanical stress-induced phenotypic switching, accumulating lipid droplets (>50% of arterial foam cells)	Demina et al. (2021)
	BRCC3-Inhibited TET2^−/−^Macrophages	Show suppressed NLRP3 inflammasome activity via blocked deubiquitylation, reversing pro-atherogenic functions	Swiatlowska et al. (2024)
	Engineered Phagocytic Macrophages	Achieve targeted plaque therapy without off-target toxicity via nanoparticle-specific delivery	Flores et al. (2019)
Myocardial infarction	Ly6C^low^Macrophages	Promote cardiac repair post-MI by enhancing survival via BDNF-TrkB signaling, improving ventricular remodeling and cardiac function	Zhang S. et al. (2024)
	Integrin α5-Dependent Macrophages	Sense ECM stiffness via α5β1-FAK mechanotransduction, polarizing toward a pro-angiogenic phenotype to orchestrate tissue repair post-MI.	Bai et al. (2022)
	Embryonic-Derived CCR2^-^Resident Macrophages	Sustain capillary density: Secrete VEGF-C, maintain vascular integrity; Promote cardiomyocyte survival via IGF-1 signaling; Proliferate locally post-MI.	Li et al. (2023)
	CD226-Dependent M1 Macrophages	Drive inflammation: Express iNOS/Mac-3; promote early injury response but impede healing via sustained inflammation	Huang et al., 2020
	CD226-KO Enhanced M2 Macrophages	Accelerate reparative processes: Express CD206; enhance collagen deposition, angiogenesis (↑α-SMA^+^myofibroblasts, ↑Ki67^+^CD31^+^ECs), and infarct stabilization	Huang et al. (2020)
	Smad3-Activated Phagocytic Macrophages	Clear apoptotic cells via upregulation of “eat-me” signals (e.g., Mfge8), preventing secondary necrosis and inflammation	Li et al. (2020)
	Smad3-Dependent Anti-Inflammatory Macrophages	Promote resolution of inflammation via IL-10/TGF-β1 secretion and support angiogenesis via VEGF production	Li et al. (2020)
	Smad3-Deficient Macrophages	Drive adverse remodeling: Impaired efferocytosis, failed anti-inflammatory transition → ↑ Cardiomyocyte apoptosis and scar expansion	Li et al. (2020)
	Mertk^+^MHC-II^lo-int^Macrophages	Drive pro-inflammatory clearance: Enhance phagocytosis of cellular debris and prime early inflammatory response post-MI	Chen et al. (2019)
	NET-Primed Pro-Inflammatory Macrophages	Ameliorate adverse remodeling: Reduce scar length/LV dilation by efficient debris clearance and limiting fibrosis	Chen et al. (2019)
	Proinflammatory Macrophages (AnxA1-deficient)	Drive adverse remodeling: Expand in ischemic myocardium, impair cardiac function, and reduce VEGF-A-mediated repair	Wei et al. (2022)
	M2 Macrophages	Secrete circUbe3a-enriched small extracellular vesicles (SEVs) that transfer pro-fibrotic signals to cardiac fibroblasts	Haider et al. (2019)
	TREM2^+^Macrophages	Execute efferocytosis and undergo immunometabolic reprogramming to produce itaconate, promoting cardiac repair	Wang et al. (2021)
	LDHA-Induced M2 Macrophages	Create a cardiac regenerative microenvironment by polarizing to an anti-inflammatory/reparative phenotype in response to cardiomyocyte-derived lactate	Gong S. et al. (2024)
Cardiac remodeling and fibrosis	IL-11-Targeted Reparative Macrophages	Attenuate pathological remodeling: Emerge after IL-11 neutralization, reducing fibrosis and inflammation	Chen Y. et al. (2022)
	CD11b^+^CD18^+^Infiltrating Macrophages	Drive vascular inflammation and dysfunction: Mediate endothelial adhesion, aortic infiltration, superoxide production, and vascular remodeling in hypertension	Humeres et al. (2019)
	CD11b-Dependent M1 Macrophages	Amplify inflammation: Secrete pro-inflammatory cytokines that exacerbate cardiomyocyte enlargement and collagen deposition	Ghazal et al. (2025)
	PAI-1-Induced M2 Macrophages	Suppress cardiac fibrosis by inhibiting TGF-β signaling and myofibroblast activation	Lin et al., 2023
	GHSR-Deficient Macrophages	Promote cardiac fibrosis by activating inflammasomes and releasing IL-18, exacerbating fibrotic remodeling	Zhang YL. et al. (2024)
	Y Chromosome-Deficient Cardiac Macrophages	Amplify age-related pathologies: Accelerate fibrotic remodeling via aberrant cytokine signaling, reducing cardiac output	Rurik et al. (2021)
	LIGHT-Induced M2 Macrophages	Drive cardiac fibrosis and AF pathogenesis: Secrete TGF-β1 to activate cardiac fibroblasts and promote collagen deposition	Baumeier et al. (2021)
	VSIG4^+^M2 Macrophages	Promote reparative fibrosis: Drive scar formation via TGF-β1/IL-10 secretion and orchestrate post-AMI inflammatory resolution	Wang et al. (2020)
	WWP2-Dysregulated Macrophages	Amplify fibrotic signaling: Promote Ccl5 expression and Ly6c^high^monocyte recruitment, enhancing collagen deposition	Sano et al. (2022)
	Spp1 Macrophages	Drive organ fibrosis: Promote fibroblast activation and collagen deposition via Spp1/Fn1/Sema3 signaling	Wu et al. (2023)
	HIF-1α-Dependent Reparative Macrophages	Restrain excessive fibrosis: Modulate fibroblast responses through hypoxia-responsive OSM release, maintaining tissue homeostasis	Wu et al. (2023)

### 3.1 Hypertension

Hypertension is a condition in which the blood pressure in the arteries is persistently elevated (Zhang et al., 2024b). Hypertension represents a significant risk factor for various cardiovascular diseases, such as heart failure, hypertensive nephropathy, aortic dissection and stroke, contributing to approximately 30% of global fatalities (Caillon et al., 2019). Human blood pressure regulation relies heavily on intricate interactions among the heart, blood vessels, and kidneys. Recent research increasingly links hypertension mechanisms to immunity and inflammation, with macrophages playing a critical role. Research conducted with animal models of salt-sensitive hypertension has revealed targeted infiltration of macrophages into the kidneys. Notably, a decrease in macrophage presence has been observed to correlate with reduced blood pressure (Fehrenbach and Mattson, 2020). Some investigations have highlighted the role of interleukin-12p35 (IL-12p35) knockout in exacerbating inflammation and raising blood pressure in mice treated with Ang II (Ye et al., 2019). In Ang II-induced hypertensive mice, studies using nod-like receptor family pyrin domain-containing 3 (NLRP3)−/− mice indicated that the absence of myeloid sirtuin-3 (SIRT3) promoted macrophage infiltration, suggesting SIRT3 as a potential therapeutic target for mitigating NLRP3-associated inflammation (Wei et al., 2021). Researchers have also identified macrophages as pivotal effectors in the antihypertensive effects of catestatin (CST), demonstrating reductions in macrophage activity *in vivo* (Ying et al., 2021). Moreover, investigations using zebrafish models of arterial hypertension induced by ionic homeostatic imbalance have underscored the importance of macrophage homeostasis in immune-targeted therapies for managing hypertension challenges (Apaydin et al., 2023). However, a retrospective clinical study noted variability in the effects of macrophage depletion on blood pressure, suggesting that differences in experimental techniques such as administration routes of macrophage-depleting agents and the extent of depletion could influence outcomes (Wenstedt et al., 2021).

In the presence of hypertension, adult cardiac muscle cells enhance cardiac function by enlarging in size and improving contraction through adaptive growth. In hypertensive conditions, the growth of cardiac muscle cells and the maintenance of cardiac function depends on resident cardiac macrophages. When macrophage-derived IGF-1 is ablated or selectively deleted by macrophages, adaptive growth of cardiomyocytes is hindered, leading to a failure in increasing cardiac mass and resulting in cardiac dysfunction (Zaman et al., 2021). Left ventricular hypertrophy (LVH) is a characteristic feature of spontaneously hypertensive rats (SHR). Application of human adipose stem cells (hADSC) in SHR models has shown a significant increase in macrophage accumulation, suggesting that hADSC might mitigate ventricular hypertrophy by modulating macrophage activity (Lee et al., 2019). In conclusion, establishing a definitive link between macrophages and the regulation of blood pressure is crucial for a comprehensive understanding of hypertension pathophysiology and could unveil new therapeutic avenues.

### 3.2 Atherosclerosis

Atherosclerosis is a chronic condition affecting the arterial wall, and is characterized by the accumulation of lipids and inflammation in the inner lining of blood vessels. It is closely associated with major clinical events such as myocardial infarction and ischemic stroke, which are leading causes of mortality (Libby et al., 2019). Elevated levels of circulating cholesterol, especially lipoprotein(a), are significant risk factors contributing to the development and progression of atherosclerosis (Tsa et al., 2023). In the process of atherosclerosis formation, reducing sources of inflammation promotes plaque regression and reduces the incidence of atherosclerotic cardiovascular disease (ASCVD). The resolution of localized inflammation largely hinges on the role of macrophages. Therefore, investigating the involvement of macrophages in atherosclerotic lesions is crucial (Bäck et al., 2019).

**FIGURE 3:**
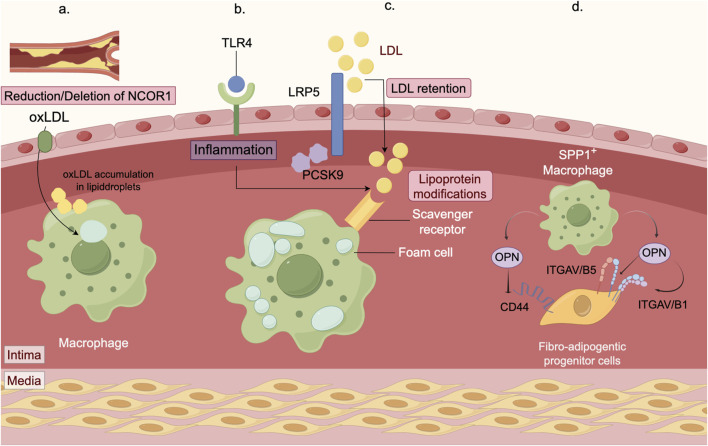
Mechanisms of macrophage in atherosclerosis. **(a)** The macrophage nuclear receptor corepressor NCOR1 mitigates atherosclerosis progression by suppressing the expression of peroxisome proliferator-activated receptor γ (PPARγ) target genes. By using myeloid-specific NCOR1 knockout mice crossed with low density lipoprotein receptor (Ldlr) knockout mice, we observed that deleting macrophage NCOR1 enhances foam cell formation, increases the expression of proinflammatory cytokines, and leads to larger atherosclerotic lesions with a more extensive necrotic core. **(b)** Macrophages are essential immune cells responsible for clearing pathogens and cellular debris. In atherosclerosis, they ingest low-density lipoprotein (LDL), transforming into foam cells that drive inflammatory responses. PCSK9 and LRP5 are involved in the formation of foam cells and the accumulation of lipids. Moreover, PCSK9 influences inflammation by modulating the TLR4 pathway. **(c)** Retained LDL and other modified LDL particles are primarily taken up in large quantities by macrophages via scavenger receptors (SRs, such as SR-A), leading to foam cell formation. The accumulation of these foam cells expands the lipid core within plaques, a key contributor to plaque vulnerability and rupture. **(d)** Macrophages are essential immune cells. In the perivascular adipose tissue (PVAT) of the coronary artery, accumulation of SPP1+ macrophages is associated with atherosclerosis and promotes fibrosis. These SPP1+ macrophages secrete osteopontin (OPN), which interacts with CD44 and integrins on fibrotic adipocyte precursors. This interaction stimulates the migration and proliferation of these precursors, thereby exacerbating PVAT fibrosis.

Studies have shown that deficiency in macrophage nuclear receptor corepressor 1 (NCOR1) increases the formation of foam cells, boosts the expression of proinflammatory cytokines, and results in larger necrotic cores within atherosclerotic lesions (Oppi et al., 2020). Additionally, both proprotein convertase subtilisin/kexin 9 (PCSK9) and LDLR-related protein 5 (LRP5) have been implicated in foam cell formation and lipid accumulation, suggesting their involvement in lipid uptake by macrophages (Badimon et al., 2021). Within macrophages, proline/serine-rich coiled-coil protein 1 (PSRC1) interacts with annexin A2 (ANXA2) to delay the onset of atherosclerosis (Pan et al., 2023). Perivascular adipose tissue (PVAT) is essential for maintaining vascular health, and PVAT dysfunction has been associated with increased atherosclerotic plaque burden. Research has highlighted the potential role of secreted phosphorylated protein 1+ (SPP1+) macrophages in PVAT in coronary atherosclerosis (Figure 3) (Fu et al., 2023).

Another potential mechanism contributing to atherosclerosis involves altering the composition of LDL particles through chemical or enzymatic modifications (Linton et al., 2023). Deamidation of LDL has been shown to increase its affinity for the scavenger receptor asialoglycoprotein receptor (ASGR), thereby enhancing uptake by intimal macrophages (Demina et al., 2021). Decreased levels of histone H3 lysine 9 trimethylation (H3K9me3) have been linked to lipid metabolism and the suppression of genes involved in regulating atherosclerosis-related factors (Swiatlowska et al., 2024). Furthermore, the deubiquitination-mediated activation of the NLRP3 inflammasome has emerged as a potential therapeutic target for preventing cardiovascular diseases associated with TET2 mutations (Yalcinkaya et al., 2023).

A defining characteristic of atherosclerotic plaques is the buildup of apoptotic cells within the necrotic core. In response to apprehensions regarding phagocyte antibody-based therapies, which may inadvertently lead to the clearance of healthy tissue and resulting in adverse reactions like anemia, researchers have developed an alternative approach: macrophage-specific nanotherapy (Flores et al., 2019). This nanotherapy has been shown to reduce plaque size in atherosclerotic apolipoprotein-deficient mice safely and effectively. Further investigation is needed to determine whether this approach, which enhances phagocytosis, contributes to plaque stabilization (Flores, 2025).

### 3.3 Myocardial infarction (MI)

Myocardial infarction (MI) is characterized as the widespread death of cardiomyocytes and acute damage to the myocardium caused by acute myocardial ischemia. Myocardial infarction (MI) occurs due to thrombosis or vascular blockage and represents the leading cause of morbidity and mortality among cardiovascular diseases (Yap, 2023). After MI, an inflammatory response is triggered primarily by infiltrating immune cells. Macrophages within the myocardium play a crucial role in modulating local immune responses in cardiac tissues following myocardial infarction (Zhang S. et al., 2024).

Some researchers have observed that an enriched environment (EE) can enhance cardiac function in mice after myocardial infarction, facilitated by Ly6C^low^ macrophages (Bai et al., 2022). Macrophages possess the ability to sense changes in the extracellular matrix environment through integrin α5, which plays a pivotal role in regulating the repair response post-myocardial infarction (Li et al., 2023). Leucine-rich repeat-containing G protein-coupled receptor 4 (Lgr4) serves as a regulator of macrophage-associated immune responses. Studies with macrophage-specific Lgr4 knockout mice (Mac-L4KO) have shown improved cardiac function and a modest reduction in infarct size, suggesting that targeting macrophage Lgr4 could be a potential therapeutic strategy for myocardial infarction (Huang et al., 2020). Additionally, research has identified a role for CD226 in infarct healing and cardiac remodeling by influencing macrophage polarization post-myocardial infarction (Li et al., 2020). Macrophage Smad3 signaling protects infarcted hearts from adverse remodeling, reduces mortality, promotes phagocytic programs, and induces anti-inflammatory shifts (Chen et al., 2019). EDIL3 acts as a natural inhibitor of neutrophil adhesion, and its absence promotes the polarization of proinflammatory macrophages, thus helping to prevent adverse cardiac remodeling (Wei et al., 2022). Furthermore, the direct influence of membrane-bound protein A1 on the polarization of cardiac macrophages towards a pro-angiogenic and reparative phenotype has also been documented (Ferraro et al., 2019).

Macrophages and fibroblasts are the primary cell types involved in the healing process after a myocardial infarction, playing essential roles in myocardial remodeling and fibrosis. Some studies suggest that therapeutic manipulation of macrophage-fibroblast interconversion could be beneficial in modulating the fibrotic response following myocardial infarction and other cardiovascular pathologies (Haider et al., 2019). Observations from an acute myocardial infarction (AMI) mouse model, which revealed the presence of macrophages and fibroblasts in the infarct zone on day 7 post-infarction, provide a foundation for potential communication between these cell types (Wang et al., 2021).

Efferocytosis and metabolic reprogramming of macrophages are both crucial for repairing myocardial infarction. A study connecting efferocytosis during myocardial repair to immune metabolism found that a deficiency in macrophage-specific TREM2 worsens cardiac dysfunction and hinders post-infarction repair (Gong S. et al., 2024). Conversely, metabolic reprogramming through lactate dehydrogenase A (LDHA) promotes cardiomyocyte proliferation by lowering reactive oxygen species (ROS) levels and encouraging M2 macrophage polarization. This suggests that targeting LDHA might be a promising strategy for enhancing cardiac repair after myocardial infarction (Chen Y. et al., 2022).

### 3.4 Cardiac remodeling and fibrosis

Following cardiac injury, the expansion and activation of fibroblasts are crucial for repair but may also drive fibrosis, pathological remodeling, and cardiac dysfunction (Humeres et al., 2019). Cardiac remodeling and fibrosis are primarily mediated by activated cardiac fibroblasts. These cells drive the pathological deposition of excessive extracellular matrix proteins within the perivascular and interstitial cardiac spaces. The activation of cardiac fibroblasts represents a pivotal process in cardiac fibrosis, initiated by a signaling cascade following injury (Ghazal et al., 2025).

**FIGURE 4:**
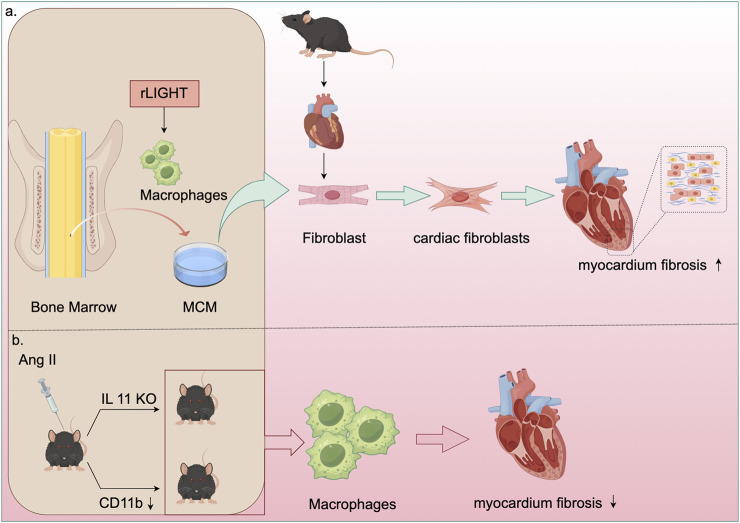
Mechanisms of macrophage in cardiac fibrosis. **(a)** Recombinant LIGHT stimulated bone marrow-derived macrophages (BMDM) to generate macrophage-conditioned medium (MCM). This MCM was then utilized to culture mouse cardiac fibroblasts, demonstrating that LIGHT controls the phenotype of myocardial fibroblasts and enhances myocardial fibrosis by promoting M2 macrophage polarization. **(b)** In AngII-treated IL-11 knockout mice, there was a reduction in macrophage infiltration, inflammatory factor expression, and fibrosis. Moreover, inhibiting CD11b enhanced macrophage adhesion and migration, thereby preventing vascular dysfunction and reducing cardiac fibrosis and adverse remodeling in AngII-infused mice.

Beyond renal and vascular inflammation, macrophages also support structural adaptations of the heart under hypertensive stress. Studies have shown that vascular hypertrophy, endothelial fibrosis, macrophage infiltration, and expression of inflammatory factors are diminished in Ang II-treated IL-11 knockout mice (Guo et al., 2021). Elevated expression of CD11b/CD18 has been observed in both patients with hypertension and mice infused with Ang II. Ablation or inhibition of CD11b has been found to inhibit macrophage adhesion and migration, thereby preventing hypertension and vascular dysfunction (Lin et al., 2023). Another study revealed that CD11b+ macrophages promote cardiomyocyte hypertrophy and fibroblast differentiation, leading to cardiac dysfunction and adverse remodeling. Thus, inhibiting CD11b could serve as an innovative strategy for managing heart failure (Figure 4) (Zhang YL. et al., 2024).

Cardiac macrophages play a significant role in the progression of cardiac fibrosis. Depletion of these macrophages results in the activation of inflammasomes, subsequently leading to cardiac dysfunction. Targeting the immune system to promote myocardial recovery and repair is a crucial goal in cardiac immunology (Rurik et al., 2021). Plasminogen activator inhibitor 1 (PAI-1) has been shown to reduce myocardial fibrosis and promote M2 macrophage polarization in inflammatory cardiomyopathy (Baumeier et al., 2021). Deficiency in the growth hormone secretagogue receptor (GHSR) worsens isoproterenol (ISO)-induced cardiac fibrosis (Wang et al., 2020). Hyperactivation of the pro-fibrotic signaling network by Y-chromosome-deficient cardiac macrophages can result in cardiac fibroblast proliferation, excessive matrix production, and reduced cardiac function (Sano et al., 2022). Tumor necrosis factor superfamily member 14 (TNFSF14, also known as LIGHT) contributes to myocardial fibrosis by influencing the phenotype of cardiac fibroblasts through M2 macrophage polarization (Figure 4) (Wu et al., 2023). V-Set and Immunoglobulin Domain Containing 4 (VSIG4), primarily expressed in intratissue and M2 macrophages, offers a potential immunomodulatory therapeutic pathway for fibrotic repair post-AMI (Wang et al., 2023). Research has indicated that WW domain-containing E3 ubiquitin protein ligase 2 (WWP2) enhances inflammatory and fibrogenic processes in cardiac macrophages, and its dysfunction inhibits cardiac fibrosis (Chen H. et al., 2022). Additionally, chemokine (C-X-C motif) ligand 4 (CXCL4) is among the most upregulated genes during the differentiation of fibrotic Spp1 macrophages, which are expanded in both chronic kidney disease and heart failure in humans (Hoeft et al., 2023). Transforming growth factor-β1 (TGF-β1) is considered essential for fibrosis development, while oncostatin-M (OSM), a member of the interleukin 6 cytokine family, directly inhibits TGF-β1-mediated activation of cardiac fibroblasts (Abe et al., 2019).

Although the role of cardiac macrophages has been extensively researched, the intricate molecular mechanisms involved in cardiac remodeling and myocardial fibrosis remain unclear and necessitate further investigation through additional studies.

### 3.5 Regulation of macrophages in cardiovascular diseases

Macrophages are the important part of the immune system *in vivo* and play the important role in many pathophysiological mechanisms and cardiovascular diseases, such as myocardial ischemia-reperfusion, myocardial infarction, atherosclerosis, etc.,. Efferocytosis is an essential part of the physiological functions carried out by macrophages. Efferocytosis of macrophages is carried out by two parts of phagocytes (Zuttion et al., 2024), namely, tissue-resident professional phagocytes including dendritic cells and macrophages and non-professional phagocytes including epithelial cells and fibroblasts. In the early embryonic development of *Drosophila*, Michael H. Raymond et al. eliminated excessive cells through efferocytosis, which could better promote the development of tissues and organs (Raymond et al., 2022). Efferocytosis can activate the pro-lysis pathway, and plays an important role in preventing inflammation ad necrosis of tissues, preventing autoimmune diseases, maintaining tissue homeostasis, and preventing chronic inflammation.

In recent years, research on the physiological basic functions and mechanisms of macrophages has received significant attention. Among them, cellular metabolism has been recognized as a dynamic regulator of macrophage activation, biology, and function. Here, we will focus on explaining how various metabolic processes influence the biological functions of macrophages.

Resolving-type macrophages predominantly utilize OXPHOS to facilitate the clearance of apoptotic cells via efferocytosis. In contrast, M2-type macrophages exhibit greater reliance on OXPHOS compared to their M1 counterparts. Notably, Wculek et al. demonstrated that caspase-11 enhances OXPHOS in both humans and mice, thereby promoting macrophage apoptosis (Wculek et al., 2023). Further studies have shown that OXPHOS can regulate their gene transcription and translation through a nuclear Atossa-Porthos axis, thereby increasing mitochondrial bioenergetics to assist in annotating cell tissue invasion in *Drosophila* 20. Uncoupling protein 2 promotes depolarization of mitochondria through inner membrane proton leakage, thereby uncoupling OXPHOS during ATP synthesis. Macrophages in mice lacking uncoupling protein 2 exhibit impaired cell burial in both *in vivo* and *in vitro*, thus demonstrating that both mitochondrial membrane potential and OXPHOS can affect efferocytosis itself.

**FIGURE 5:**
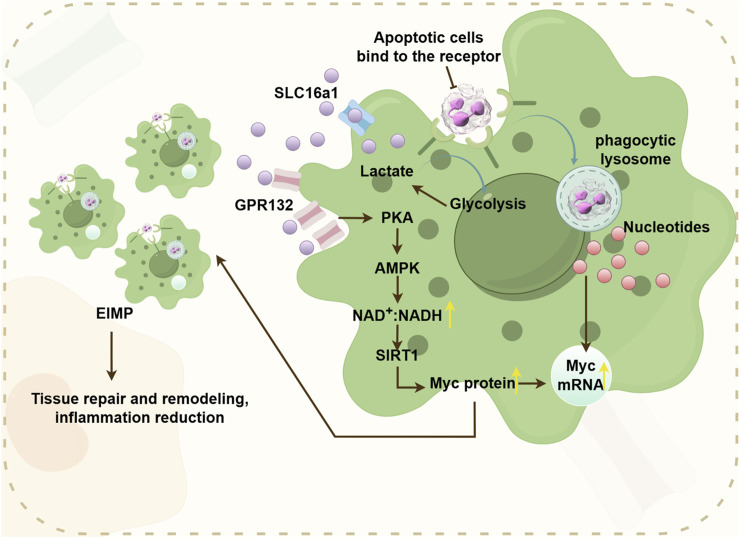
The pathway where efferocytosis-induced lactate (EIL) promotes EIMP, supports tissue repair and reduces inflammation. Efferocytosis-induced glycolysis stimulates the secretion of lactate, which activates the cell surface receptor GPR132. GPR132 then transduces signals through the PKA-AMPK-NAD + pathway. This pathway deacetylates Myc proteins, enhancing their stability. Consequently, this cascade promotes EIMP, facilitating tissue repair and reducing inflammation.

Glycolysis plays a crucial role in the macrophage efferocytosis. M1-type macrophages exhibit a close association with glycolysis, allowing them to dynamically switch between oxidative phosphorylation under aerobic conditions and glycolysis under anaerobic conditions in response to microenvironmental stimuli. In 2023, Schilperoort et al. discovered that efferocytosis triggers transient activation of glycolysis in macrophages. They also discovered that PFKFB2-mediated glycolysis upregulates lactate-mediated cytosolic sequestration receptors, thereby enhancing sustained efferocytosis. Additionally, autocrine and paracrine secretion of metabolic pathways contribute to sustaining prolonged efferocytosis (Schilperoort et al., 2023). Efferocytosis facilitated by maresin conjugates in tissue regeneration (MCTRs), becomes active through glycolytic metaboism, and relies on ras-related C3 botulinum toxin substrate 1(Rac1) signaling (Koenis et al., 2024). Efferocytosis can promote the decomposition of glucose, thereby generating lactic acid. Cell burial can stabilize Myc proteins and promote macrophage proliferation (EIMP) by integrating with nucleotide pathways through glycolycle-induced lactic acid (EIL) -mediated pathways. The lactate receptor GPR132 can transmit EIL signals through the PKA - AMPK - NAD^+^ -SIRT1 pathway and drive EIMP through deacetylation of Myc protein. When EIMP is damaged, it will cause impaired efferocytosis, which in turn leads to an excessive accumulation of apoptotic cells in the tissue. Meanwhile, researchers also found that excessive lactic acid can inhibit EIMP, which also indicates that the lactic acid concentration produced by glycolysis induced by efferocytosis may be the optimal concentration for EIMP (Nature Metabolism, 2023). Lactic acid can not only induce EIMP through the lactic acid receptor GPR123, but also affect macrophages by entering the TCA cycle. Lactic acid serves as an important fuel in the TCA cycle in many tissues. The downregulation of lactic acid transport proteins also weakens OXPHOS in macrophages, which indirectly proves that lactic acid is an important source of OXPHOS. On the other hand, lactic acid can also conduct signal transduction through the lactic acid receptor GPR81, which transmits signals to surrounding cells to exert its function (Figure 5) (Nature Metabolism, 2023).

Amino acids serve as the basic building blocks of proteins. Both M1 and M2 macrophage types can modulate specific pathways of their response by regulating amino acid metabolism. The sustained process of efferocytosis involves the metabolism of arginine and ornithine derived from apoptotic cells into putrescine (Yurdagul et al., 2020). Tryptophan plays a role in immune tolerance in peripheral blood through the action of indoleamine 2,3-dioxygenase 1 (IDO1). Glutamine can be induced by glutaminase and is highly expressed in M2-like phenotypes, where its expression is influenced by IL-10 signaling (Palmieri et al., 2017). The enzyme IDO1 catalyzes the conversion of L-tryptophan to kynurenine (KYN) as part of the KYN pathway, which activates the aryl-hydrocarbon receptor (AhR) through autocrine or paracrine secretion. The IDO1-KYN-AhR pathway enhances sustained efferocytosis (Salminen, 2022).

The process of efferocytosis involving nucleotides has been identified as resolving tissue damage by promoting EIMP, which depends on the phagolysosomal degradation of apoptotic cells (AC) and further activation of MerTK. Macrophage proliferation requires the release of oligonucleotides produced from AC within the phagolysosome, which activates mTORC2. Additionally, it has been observed that macrophages undergoing EIMP are efficient efferocytes capable of producing transforming growth factor-β (TGFβ) and interleukin-10 (IL-10) *in vitro* (Gerlach et al., 2021).

Lipid synthesis plays a critical role in macrophage function. Upon microbial stimulation, macrophages can initiate *de novo* lipogenesis (DNL) and activate the nuclear factor kappa-B (NF-κB) signaling pathway, influencing the transcriptional regulation of lipid synthesis. Upon exposure to inflammatory stimuli, macrophages increase their uptake of free fatty acids (FAs) and lipoproteins. Mitochondrial fatty acid oxidation plays a crucial role in facilitating several mechanisms through which IL-4 activates M2-related macrophages. Cholesterol crystals contribute to the inflammatory vesicle pathway in localized macrophages, leading to the production of IL-1β. Additionally, 25-hydroxycholesterol (25HC), an intermediate in cholesterol metabolism, mitigates cardiac lipotoxicity and offers protection against cardiomyopathy induced by type 2 diabetes (Zhang et al., 2024e).

## 4 Therapeutic potential of macrophages in cardiovascular diseases

Multiple studies have identified a close association between macrophages and NF-κB, a transcription factor that regulates various biological responses (Zhang et al., 2023). Through NF-κB signaling, macrophages can be polarized and activated, playing a crucial role in the inflammatory response and tissue repair in various cardiovascular diseases (Liu et al., 2023). NF-κB activation enhances STING signaling by regulating microtubule-mediated STING transport (Zhang et al., 2023). Losartan and angiotensin-(1–7) can modulate macrophages via NF-κB and mitogen-activated protein kinase (MAPK) pathways, inducing a shift from M1-type to M2-type macrophages. This process helps attenuate inflammatory responses, maintain mitochondrial dynamics homeostasis in cardiomyocytes, reduce oxidative stress and apoptosis in cardiomyocytes, and potentially treat sepsis-induced cardiomyopathy (Chen X. et al., 2022; Chen et al., 2023). Exosomes from bone marrow mesenchymal stem cells also promote M2-type polarization by activating the NF-κB pathway, aiding in inflammation resolution and reducing myocardial infarction severity (Ouyang et al., 2024). Additionally, TNFSF13, a paracrine factor upregulated in M2 macrophages, can promote proliferation and fibrotic changes in cardiac endothelial cells and myocardial fibers through NF-κB and Akt pathways, providing potential targets and insights for treating cardiovascular diseases (Chen et al., 2024).

Additionally, signals from apoptotic cells, such as ferroptotic cells, can induce macrophage conversion from M1 to M2 via exosomal cargos. This process is typically mediated by extracellular vesicles (EVs) that release “Find-Me” and “Eat-me” intercellular signals. These signals undergo efferocytosis interactions, regulating antigen presentation and facilitating crosstalk with immune cells, repair of damaged tissues, and maintenance of homeostasis. Recent studies utilizing nanoparticle tracking analysis, RT-qPCR, protein blotting, transmission electron microscopy, and immunohistochemistry have revealed that M2 macrophage-derived exosomes and their carriers, including miR-146a-5p, can shorten the duration of action potentials resulting from rapid pacing. This effect is mediated by the NF-κB/signal transduction and transcriptional activators (STAT3) signaling pathway expressed by Kca3.1, which inhibits the secretion of IL-1β and reduces HL-1 in pacemaker cells, presenting an innovative therapeutic approach for atrial fibrillation (Chen et al., 2024).

Furthermore, the regulation of signal transduction and expression of transcriptional activators may be connected to M1-type macrophage polarization (Zhang et al., 2018). MSCNIC-exo can enhance macrophage M2 polarization by upregulating miR-125a-5p, which targets the TRAF6/IRF5 signaling pathway, significantly improving cardiac repair (Gong ZT. et al., 2024). These discoveries propose innovative and viable therapeutic strategies for the future.

Targeted therapeutic microRNAs (miRNAs) have gained significant attention in recent years. These can be delivered into the cytoplasm using platelet membrane-modified EVs to facilitate the transition from M1-type to M2-type macrophages, thereby regulating the immune microenvironment (Li et al., 2022). In 2024, Pei et al. developed a targeted therapeutic agent incorporating a cardiotargeting peptide (CTP) enriched with viral macrophage inflammatory protein-II (vMIP-II) and platelet membrane (PM)-engineered M2 EVs. This innovation improved the delivery of EV “cargo” to cardiac tissue and enhanced the targeting ability of M2 EVs. Consequently, it reduced chemokine receptor expression and co-regulated M1-type macrophages in the inflammatory microenvironment with M2 EVs, showing potential for immunomodulatory therapy in cardiovascular disease (Pei et al., 2024). Additionally, a recent development in hydrogel-encapsulated photoresponsive upconverted cyanobacteria nanotechnology capsules (UCCy@Gel) has shown promise. These capsules inhibit M1 macrophage polarization by releasing light and oxygen under 980 nm near-infrared irradiation, reducing the expression of pro-inflammatory cytokines IL-6 and tumor necrosis factor-α. This approach offers hypoxia prophylaxis and oxygen therapy with a single injection (Liu et al., 2022).

**FIGURE 6:**
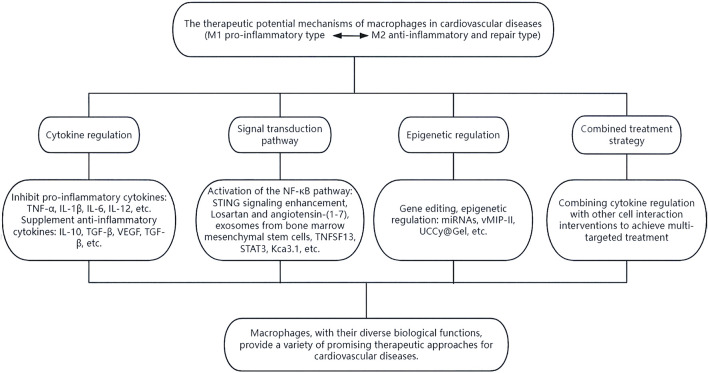
The therapeutic potential of macrophages. Macrophages can exert their diverse biological functions through various means such as cytokine regulation, signal transduction pathways, epigenetic regulation, and combined treatment strategies, thereby providing multiple promising therapeutic approaches for cardiovascular diseases.

Lactate enhances inflammation in adipose macrophages by targeting PHD2 (Feng et al., 2022). Macrophages can potentially impede cardiomyocyte senescence by restricting the transfer of adipocyte mitochondria into cardiomyocytes (Borcherding et al., 2022). The IL6/ADPN/HMGB1 axis is a potential target for regulating communication between adipocytes and macrophages, promoting M2 polarization in macrophages (Zheng et al., 2024).Reducing macrophage infiltration and the expression of proinflammatory cytokines (IL-1β and IL-6) during myocardial infarction can be achieved by downregulating TRIM21, thereby preventing atrial inflammation and post-infarction remodeling (Figure 6) (Liu et al., 2023).

In summary, macrophages present a variety of promising therapeutic avenues for cardiovascular diseases through their diverse biological functions, which necessitate further research and exploration.

## 5 Current limitations and future direction

Our understanding of macrophages and their metabolism in human tissues and cells remains limited, because most of the research data on the mechanism of macrophages in cardiovascular diseases come from animal models, such as mice and zebrafish, as well as *in vitro* models, such as primary cells, cell lines like H9C2, or cardiomyocytes differentiated from human induced pluripotent stem cells. More real-world clinical studies are needed to further determine the mode and mechanism of macrophages’ role in cardiovascular diseases. Furthermore, several studies lack large sample sizes and long-term cohort follow-up, which further constrains our knowledge. Challenges persist in the field of nanotechnology for drug delivery targeting macrophages. It is crucial to carefully consider factors like size, shape, charge, class, and functional modifications of nanomedicine to improve pharmacokinetics, enhance targeting precision, and increase solubility. Another challenge is the potential toxicity of immunotherapy affecting normal tissues and its low response rates. These factors need careful consideration in the advancement and implementation of nanomedicine technologies. Additionally, the complex preparation processes and the requirement for high product stability pose hurdles for translational research on immune-based nanomedicines centered around macrophages. To address these challenges, there is a growing need to develop various small molecule reagents with diverse functions, such as short interfering RNAs (siRNAs) or compounds, to replace large molecule antibodies. This approach could unlock the full potential of nanotechnology-based therapies in the treatment of cardiovascular diseases and beyond.

## 6 Conclusion and prospects

In recent years, advancements in scientific and technological methods have deepened our understanding of macrophage phenotypes and functions. Macrophages are pivotal in cardiovascular diseases, crucially involved in immune regulation and inflammation-related pathogenesis. The link between macrophages and blood pressure regulation is increasingly recognized as significant in pathophysiology. Exploring macrophage-associated immune and inflammatory mechanisms holds promise for advancing immunotherapy in hypertension. Animal models like mice and zebrafish provide valuable platforms for studying macrophage actions in hypertension. Macrophages also play a central role in the pathogenesis of atherosclerosis, where modifications of low-density lipoprotein (LDL) can influence macrophage function and contribute to the progression of disease. In myocardial infarction, efferocytosis and metabolic reprogramming of macrophages are critical processes that could potentially guide future therapeutic strategies. Macrophages exhibit a complex role in cardiac fibrosis and remodeling, influenced by various transcription factors, signal transduction pathways, and proteases. Further research into these molecular mechanisms is essential for deeper insights. As experimental technologies evolve, larger sample sizes will enable the analysis and identification of different macrophage subpopulations, activation mechanisms, roles, and mediators. This broader understanding promises to advance the development of immune-related therapies in diverse cardiovascular conditions.

In summary, macrophages have emerged as promising therapeutic targets for a range of cardiovascular diseases. Several new macrophage targets have been identified in pre-clinical studies. However, these targets require further validation through additional clinical research, with the potential to evolve into innovative strategies for clinical treatment.
